# Refined Qingkailing Protects MCAO Mice from Endoplasmic Reticulum Stress-Induced Apoptosis with a Broad Time Window

**DOI:** 10.1155/2012/567872

**Published:** 2012-03-22

**Authors:** Fafeng Cheng, Xianggen Zhong, Yi Lu, Xueqian Wang, Wenting Song, Shaoying Guo, Xiaotong Wang, Dantong Liu, Qingguo Wang

**Affiliations:** ^1^College of Basic Medicine, Beijing University of Chinese Medicine, Beijing 100029, China; ^2^Xiyuan Hospital, China Academy of Chinese Medical Sciences, Beijing 100091, China

## Abstract

In the current study, we are investigating effect of refined QKL on ischemia-reperfusion-induced brain injury in mice. *Methods. *Mice were employed to induce ischemia-reperfusion injury of brain by middle cerebral artery occlusion (MCAO). RQKL solution was administered with different doses (0, 1.5, 3, and 6 mL/kg body weight) at the same time of onset of ischemia, and with the dose of 1.5 mL/kg at different time points (0, 1.5, 3, 6, and 9 h after MCAO). Neurological function and brain infarction were examined and cell apoptosis and ROS at prefrontal cortex were evaluated 24 h after MCAO, and western blot and intracellular calcium were also researched, respectively. *Results. *RQKL of all doses can improve neurological function and decrease brain infarction, and it performed significant effect in 0, 1.5, 3, and 6 h groups. Moreover, RQKL was able to reduce apoptotic process by reduction of caspase-3 expression, or restraint of eIF2a phosphorylation and caspase-12 activation. It was also able to reduce ROS and modulate intracellular calcium in the brain. *Conclusion.* RQKL can prevent ischemic-induced brain injury with a time window of 6 h, and its mechanism might be related to suppress ER stress-mediated apoptotic signaling.

## 1. Introduction

Ischemic stroke is a life-threatening disease featured by high morbidity and mortality. Investigating new drugs is a hotspot and the difficulty of ischemic stroke research. The committee of the Stroke Therapy Academic Industry Roundtable proposed that studies of drugs for brain ischemia should pay attention to their pharmaceutical window [1, 2]. In clinical practice, treatment for stroke patients is always delayed after onset, and the time at which treatment is administered is highly correlated with therapeutic effect.

Qingkailing (QKL) injection was originally prepared by the Beijing University of Chinese Medicine in the 1970s, by modifying a traditional Chinese medicine, Angongniuhuang pills, composed of *Radix isatidis*, *Flos Lonicerae*, *Concha Margaritifera Usta*, baicalin, *Fructus gardeniae*, cholic acid, hyodeoxycholic acid, and *Cornu Bubali* [3]. It has been extensively used to treat the acute stages of cerebrovascular disease and has performed excellently in improving neurological function [4]. Animal experiments have shown that QKL injection can promote endothelial nitric oxide synthase expression, reduce calcium overload, regulate matrix metallopeptidase 9 expression, and inhibit inflammation in the murine model of cerebral ischemia/reperfusion [5–8]. However, QKL is confronted with handicaps from drug safety regulations in recent years, which has severely embarrassed its clinical application [9]. QKL being composed of complex components is the central problem of quality stabilization and clinic safety. Based on this, we developed refined Qingkailing (RQKL), aimed at acute ischemic stroke.

**Figure 1:**
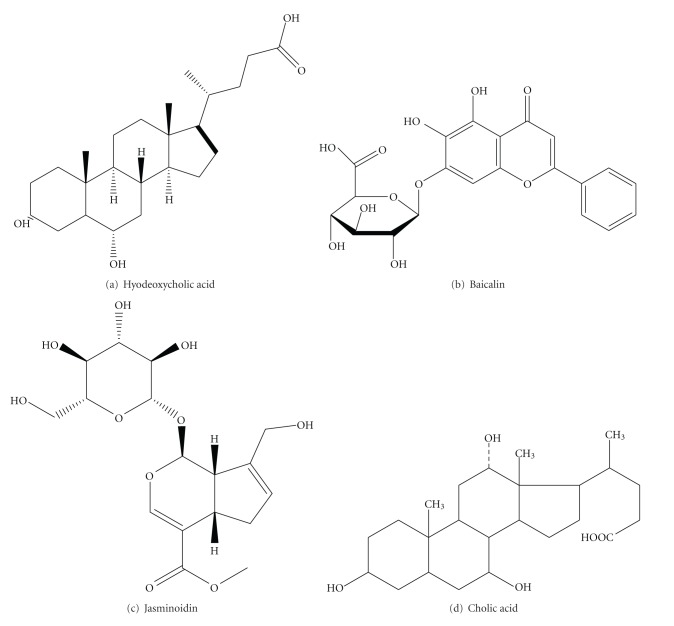
Molecular structure of the four components of RQKL.

Refined Qingkailing (RQKL) injection consists of cholic acid, hyodeoxycholic acid, baicalin, and jasminoidin (Figure 1). Baicalin is derived from the dried root of *Scutellaria baicalensis* Georgi which is named as Huang Qin in traditional Chinese medicine (TCM), and jasminoidin is derived from the dried fruit of* Gardenia jasminoides* Ellis which is named as Zhi Zi in TCM. Both herbs provide excellent antioxidative and anti-inflammation effects [10–13]. Cholic acid and hyodeoxycholic acid are both bile acids and have neuron-protective effects [14, 15]. Furthermore, the combination of the four components can significantly improve their effectiveness [16].

The endoplasmic reticulum (ER) regulates protein synthesis, protein folding and trafficking, cellular responses to stress, and intracellular calcium levels [17]. The conditions that impair the function of the ER, designated “ER stress”, can lead to an accumulation of unfolded proteins in the ER lumen [18]. However, if ER stress is too severe, the unfolded protein response ultimately initiates an apoptotic pathway [19]. ER stress-induced cell death has been shown to involve the activation of caspase-12 [20, 21], while another component of the ER stress-mediated apoptotic pathway is elf-2a [22]. Several studies have shown that cerebral ischemia is a pathological ER stressor, which can trigger the shutdown of protein translation and apoptosis, suggesting that ER plays an important role in cerebral ischemia [23, 24]. Thus, reducing ER stress may provide a therapeutic way to block the pathological process induced by cerebral ischemia [18, 24].

This paper is designed to explain the dose effect and therapeutic time window of RQKL on MCAO rodents, as well as to elaborate intervention effect of RQKL on neuron apoptosis due to ER stress after cerebral ischemia.

## 2. Materials and Methods

### 2.1. Animals

We used 154 healthy, male, Kunming mice weighing 25–28 g, and 90 healthy, male, C57BL/6 mice weighing 25–30 g, purchased from Vital River Laboratories, Beijing, China (no. SCXK (Beijing) 2006-0009) and housed in the Central Laboratory, Beijing University of Chinese Medicine on a 12 h light: dark cycle at 25 ± 1°C, with relative humidity of 40–60% and automatic day-night rhythm, the mice had free access to standard lab chow and tap water. Procedures involving animals and their care were conducted in compliance with institutional guidelines in accordance with NIH Guide for the Care and Use of Laboratory Animals, NIH publication no. 85-23, 1985.

### 2.2. Drugs

QKL injection, composed of cholic acid, *Concha Margaritifera Usta* (powder), hyodeoxycholic acid, *Fructus Gardeniae*, *Cornu Bubali* (powder), *Radix Isatidis*, baicalin, and *Flos Lonicerae* [25], was purchased from the Pharmaceutical Factory of Beijing University of Chinese Medicine (no. 813204A). RQKL injection composed of cholic acid, hyodeoxycholic acid, baicalin, and jasminoidin (Figure 1). Proportion and concentration of 4 compositions is consistent with QKL injection. RQKL injection is provided by Prof. Shouying Du, affiliated to School of Chinese Materia Medica, Beijing University of Chinese Medicine.

### 2.3. MCAO Model Establishment

Mice were anesthetized with 4% chloral hydrate (350 mg/kg) and kept under heating lamp to maintain the core body temperature at 36.5 ± 0.5°C. Under a dissecting microscope (SXE-1, Shanghai Precision Instrument, Shanghai, China), the right common carotid artery, internal carotid artery, and external carotid artery were carefully exposed, and the external carotid artery was coagulated distal to the bifurcation. A 0.16 mm diameter nylon filament (tip diameter 0.20 ± 0.01 mm; Beijing Sunbio Biotech, Beijing, China) was inserted through the external carotid artery stump and gently advanced 10 mm to occlude the origin of the middle cerebral artery [26, 27]. The body temperature of the animals was maintained at 37°C. The filament was removed after 1.5 h. Postoperatively, the mice were housed separately. Neurological function was evaluated when the mice were awake [28]; those with scores less than 2 were excluded.

### 2.4. Grouping of Experimental Animals and RQKL Injection

In the dose-effect experiments, high, moderate, low dose RQKL injection and QKL injection and model groups were injected with 6, 3, 1.5 mL/kg RQKL and 3 mL/kg QKL or equal volume of normal saline, respectively, via the tail veins [7]. Both injections were diluted in saline with different concentrations and the final dose injected to each animal was 9 mL/kg. The first injection was performed immediately after model establishment, followed by administration after 4 h, and once every 12 h thereafter.

For time window experiments, the model group was injected with normal saline, and each RQKL group with RQKL (3 mL/kg) diluted using normal saline, via the tail vein. In model and 0 h groups mice, were first injected simultaneously with the middle cerebral artery, was occluding. The other groups received RQKL injections at 1.5, 3, 6, 9 h after MCAO, followed by a second injection after 4 h, and every 12 h thereafter [29], Figure 3(A).

### 2.5. Assessment of Neurological Function after Focal Cerebral Ischemia/Reperfusion Using Clark Scores

Mice neurological function was evaluated using a blind method 24 h after model establishment. Clark scores [28] include focal and general neurological function, which reflect ischemia foci-induced neurological function injury and general function, respectively. The focal neurological function was scored from 0–28, and the general function ranged from 0–32. Normal mice had a score of 0. High scores reflect severe neurological functional injury.

### 2.6. Infarct Volume Assessment

Following neurological function evaluation, mice were sacrificed, and the brain was harvested for TTC staining (Nanjing Greensynthesis Biochemical Co., Ltd., Nanjing, Jiangsu, China). The percent of infarct volume out of the entire brain represented the degree of cerebral infarction. Serial coronal sections (1 mm thickness) were prepared and soaked in 2% TTC phosphate buffer at 37°C for 10 minutes in the dark. Normal brain tissues were stained red, while infarct tissues were not stained (white). The sections were soaked in 4% paraformaldehyde phosphate buffer for 30 minutes, arranged in order and scanned (Tsinghua Unisplendour A688, Xi'an, China). Areas of red and white staining were measured using a computer color multimedia image analysis system (Image-Pro Plus6.0, Media Cybernetics, Wyoming, USA). The percent of infarction is given by the equation: %Infarct volume = Infarct volume/Total volume of slice × 100 [30, 31].

### 2.7. TUNEL Staining

After 24 h of recovery, the animals were euthanatized and the brain was rapidly removed, frozen, and cut into 20 *μ*m slices. Terminal deoxynucleotidyl transferase dUTP nick end labeling (TUNEL) staining was performed using a kit for programmed cell death (In Situ Cell Death Detection Kit, TMR Red, Roche, USA) according to the manufacturer's directions [32]. Sections of prefrontal cortex were collected. Five areas of each section were examined by fluorescence microscope (ZEISS, LSM510 meta, Germany) in the prefrontal cortex of the ischemic hemisphere and TUNEL-positive cells were quantified [18].

### 2.8. Measurement of ROS Generation

Brain reactive oxygen species (ROS) production was determined using dihydroethidium (DHE) microfluorography [33]. DHE is a cell permeable dye, which can be oxidized into ethidium and other products by superoxide [33, 34]. Animals were sacrificed at 24 h after MCAO, and the brains were removed, frozen, and sectioned (20 *μ*m thickness) on a cryostat. Sections of prefrontal cortex were collected. A ROS fluorescence detection kit (Genmed, Wyoming, USA) was used. DHE solution was superfused on the brain sections for 60 minutes and fluorescence intensity was detected by fluorescence microscopy (ZEISS, LSM510 meta, Germany). The fluorescence intensities of five different fields of the brain section were averaged and expressed as relative fluorescence units (RFU) [35].

### 2.9. Western Blot

Mice were sacrificed after 24 h of cerebral ischemia, the forehead cortex was collected and homogenated in an ice bath. Proteins were separated on sodium dodecyl sulfate polyacrylamide gel electropheresis and transferred to a nitrocellulose membrane. Blots were blocked with 5% nonfat dry milk in phosphate buffered saline, pH 7.6, with 0.1% Tween-20 buffer and then incubated with antiphospho-eIF2*α* (Ser51) polyclonal antibody (Cell Signaling Technology Inc., Tokyo, Japan), anti-caspase12 polyclonal antibody (Cell Signaling Technology Inc., Tokyo, Japan), or anti-caspase3 (8G10) polyclonal antibody (Cell Signaling Technology Inc., Tokyo, Japan), subsequently incubated with secondary anti-rabbit antibody conjugated with horseradish peroxidase. Finally, membranes were processed for detection using the ECL system.

### 2.10. Intracellular Calcium Measurement

Intracellular calcium concentration was measured by flow cytometry using Fluo-3AM fluorescence as described previously [36, 37]. Mice were sacrificed 2 h after middle cerebral artery occlusion or 10 h reperfusion after 2 h of occlusion. Forehead cortex and hippocampus were separated in ice bath and prepared into cell suspension. Cell density was adjusted with D-Hanks solution to about 1 × 10^6^. Suspension was incubated at 37 for 10 minutes, then 2 *μ*L/mL Fluo-3AM dye working solution was added and mixed to uniform. Mixed solution was incubated at 37°C for 40 minutes in dark place. Flow cytometry (FACS Calibur, Becton Dickinson, USA) analysis was performed with excitation wavelength 488 nm and emission wavelength 528 nm. The results are expressed as mean fluorescence intensity (MFI) [37, 38].

### 2.11. Statistical Analysis

Data were analyzed using SPSS 16.0 (SPSS, Chicago, IL, USA). One-way analysis of variance was used followed by post hoc analysis for significance with the Student-Newman-Keuls multiple comparison test. All values are expressed as mean ± SEM. A value of *P* < 0.05 was considered statistically significant.

## 3. Results

### 3.1. Dose-Response of RQKL for Focal Cerebral Ischemia/Reperfusion

#### 3.1.1. Dose-Response Effects on Infarct Volume

**Figure 2:**
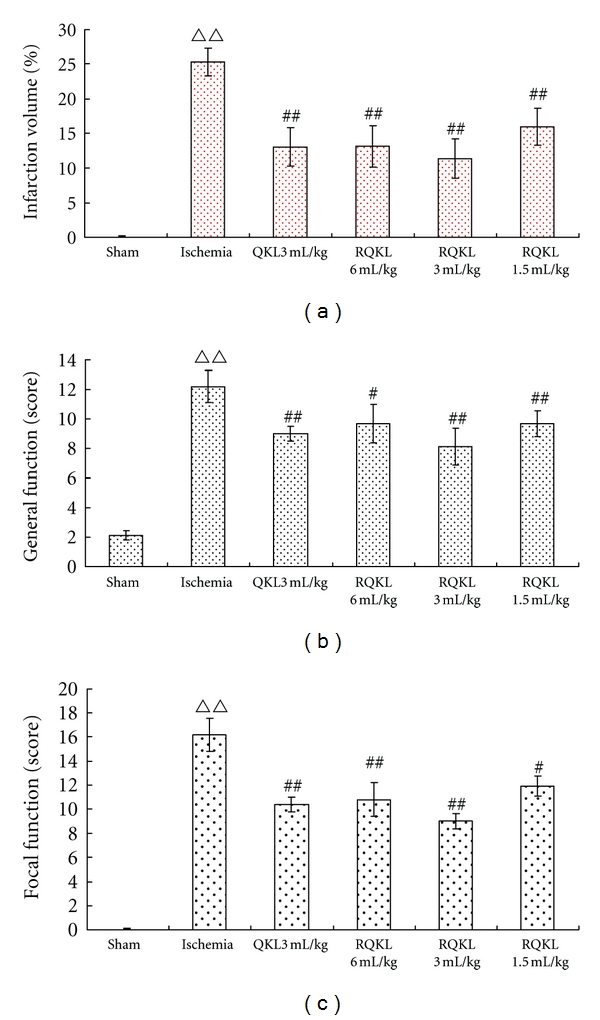
Effect of different doses of RQKL on infarction volume and neurological function of MCAO mouse. Five brain coronal sections, 2 mm thick, were selected for tetrazolium chloride staining. The infarct volume was quantified as a percentage of total volume, with large infarcts representing more severe injury. The percent of infarct volume was significantly less in RQKL injection groups compared with model group (a). RQKL injection at different doses significantly improve focal and general neurological function (b, c). The scores of focal neurological function and general neurological function were 0 in normal mice. High scores represent severe injury. ^ΔΔ^ *P* < 0.01, versus model group, ^#^ *P* < 0.05, ^##^ *P* < 0.01, versus model group. Data are expressed as mean ± SEM, *n* = 10.

Mice with focal cerebral ischemia/reperfusion were injected with different doses of RQKL. Infarct foci were evident in the brain of mice at 24 h after ischemia/reperfusion. Compared to model group, the infarction size was reduced by 49%, 48%, 55% and 37% (*P* < 0.01) in QKL and high, moderate, and low dose RQKL injection groups, respectively (Figure 2(a)).

#### 3.1.2. Dose-Response Effects on Neurological Function

Prior to MCAO, scores of focal neurological function and general neurological function were 0. Mice developed neurological functional injury 24 h after MCAO; however, QKL and all doses of RQKL ameliorated this injury compared with the model group (*P* < 0.05 or *P* < 0.01). The results are consistent with our preliminary experiments [29]. The groups receiving the moderate QKL and RQKL dose (3 mL/kg) exhibited the greatest improvement (*P* < 0.01). Moderate and high dose RQKL injection was able to restore focal neurological function (*P* < 0.01); however, the effect of low dose group was relatively poor (*P* < 0.05), Figures 2(b) and 2(c).

### 3.2. The Therapeutic Time Window of RQKL

#### 3.2.1. Cerebral Infarct Volume after Treatment with RQKL at 0, 1.5, 3, 6, 9 h after Ischemia

**Figure 3:**
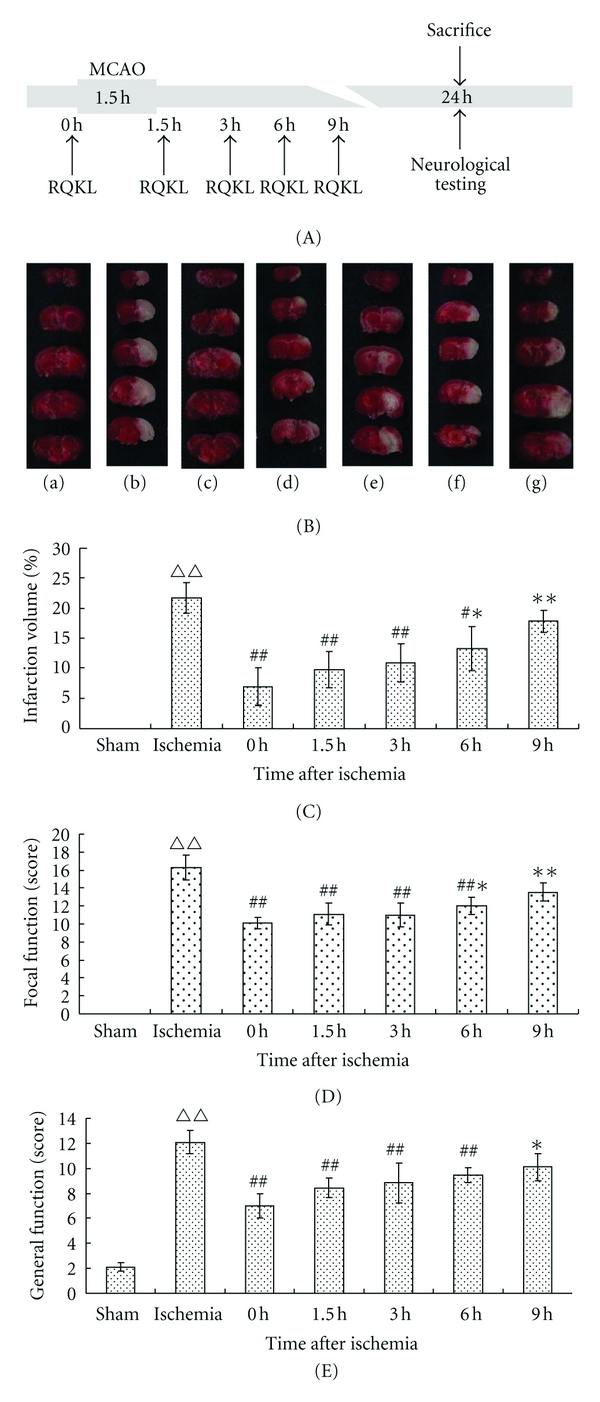
Effects of *RQKL *injection at different time points in mice undergoing middle cerebral artery occlusion. Flow chart of time window experiment of RQKL (A). Cerebral infarct volume (B, C) and neurological function scores (D, E) were evaluated 24 h after brain ischemia. Five brain coronal sections, 2 mm thick, were selected for tetrazolium chloride staining. Red stain represents normal tissues; white represents the infarct region (B). In panels (D) and (E), high scores represent serious injury. RQKL injection at 0, 1.5, 3, or 6 h after infarction significantly reduced infarct volume and improved focal and general neurological function, but 9 h group had no effects. ^ΔΔ^ *P* < 0.01, versus model group, ^#^ *P* < 0.05, ^##^ *P* < 0.01, versus model group. Data are expressed as mean ± SEM, the numbers of each group were 10, 11, 12, 9, 9, 12, 9, respectively.

In preliminary experiment, we found that QKL had a wide therapeutic time window [29]. In this study, RQKL also showed a broad time window. Injection of 3 mL/kg RQKL was most effective at reducing infarct volume and improving neurological function, so this dose was used in time window experiments. RQKL injection at 0, 1.5, 3, and 6 h after ischemia significantly reduced cerebral infarct volume (*P* < 0.01 or *P* < 0.05). The largest reduction of infarct volume (68%) was in the 0 h group compared with model group (*P* < 0.01). Percent reduction of infarct volume gradually decreased with increasing delay before QKL injection (infarct size: 55%, 50%, 39% in the 1.5, 3, and 6 h groups, resp.). However, 9 h group reduced cerebral infarct volume 18% than model group, but with no significance (*P* > 0.05) (Figures 3(A) and 3(B)).

#### 3.2.2. Neurological Function Scores after Treatment with RQKL at 0, 1.5, 3, 6, 9 h after Ischemia

Consistent with dose-effect experiments, the general and focal neurological functions were significantly improved in the 0 h group (*P* < 0.01). In other treatment groups, RQKL injected at 1.5, 3, and 6 h after MCAO significantly enhanced both general and focal neurological functions (*P* < 0.01), but administration at 9 h was not effective. However, with the first treating time delay, the effects gradually decreased. The focal neurological function of 6 and 9 h groups were significantly lower than 0 h group (*P* < 0.05, *P* < 0.01, resp.), while general neurological function of 9 h group was significantly lower than 0 h group (*P* < 0.05) (Figures 3(C) and 3(D)).

#### 3.2.3. Effects of RQKL on Neurons Apoptosis

**Figure 4:**
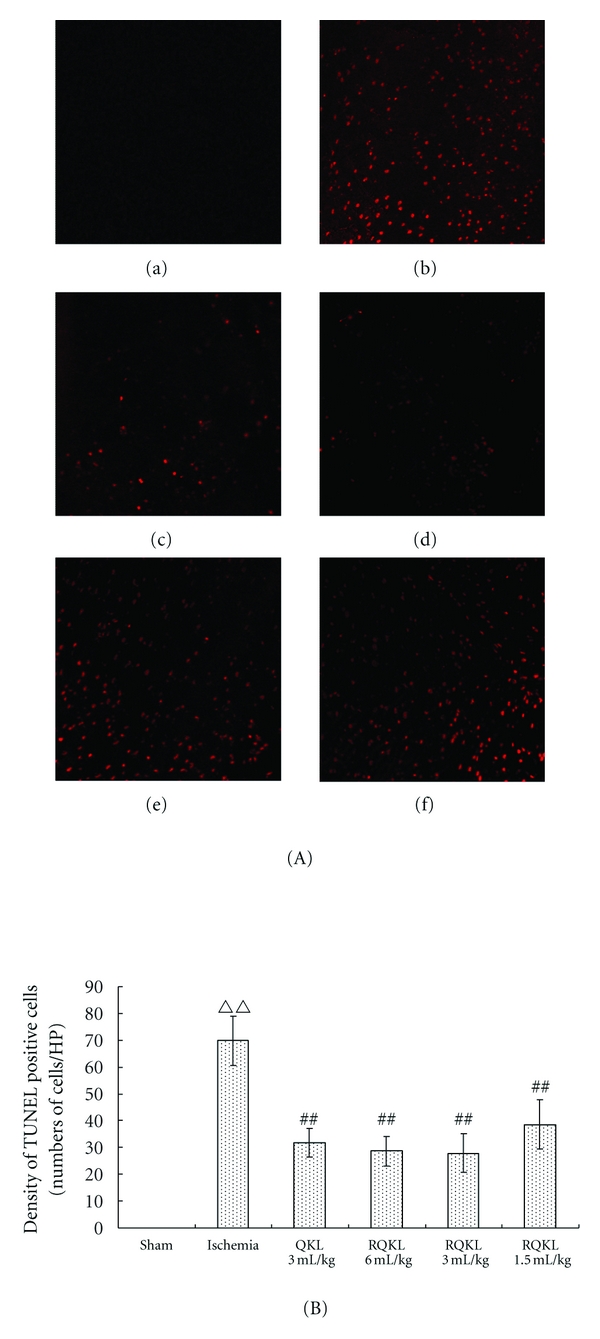
Effect of* RQKL* injection on cell apoptosis in prefrontal cortex of mice (TUNEL staining). After middle cerebral artery occlusion for 24 h, apoptotic cells were detected in the prefrontal cortex ((A), ×400). Apoptotic cells were labeled with red fluorescence. (a) sham, (b) ischemia, c-ischemia+QKL3ml/kg, d-ischemia+RQKL 6 mL/kg, (e) ischemia+RQKL3ml/kg, (f) ischemia+RQKL1.5 mL/kg. Five animals were selected from each group; three sections were selected from each site; and five 400-fold fields of view were randomly selected from each section to quantify the mean of positive cells. Results are expressed as mean ± SEM (B). ^ΔΔ^ *P* < 0.01, versus sham-surgery group; ^##^ *P* < 0.01, versus model group.

Cellular apoptosis is an important mechanism of nerve injury after brain ischemia/reperfusion [18]. The TUNEL method was used to investigate whether an antiapoptotic effect was involved in the neuroprotection by RQKL. Few apoptotic cells were observed in brain tissues of sham mice. A large number of TUNEL-positive neurons were observed in the prefrontal cortex 24 h after ischemia. Following QKL (3 mL/kg) or RQKL (6, 3 and 1.5 mL/kg) injections, the number of TUNEL-positive neurons was reduced significantly (Figures 4(a), 4(b), and 4(c)). This showed that RQKL reduced the number of apoptotic cells in brains of MCAO mice. Meanwhile, the antiapoptosis effect of RQKL showed dosedependence.

#### 3.2.4. Effects of RQKL on Protein Level of Caspase-3, Pro-Caspase12, and P-eIF2*α*

**Figure 5:**
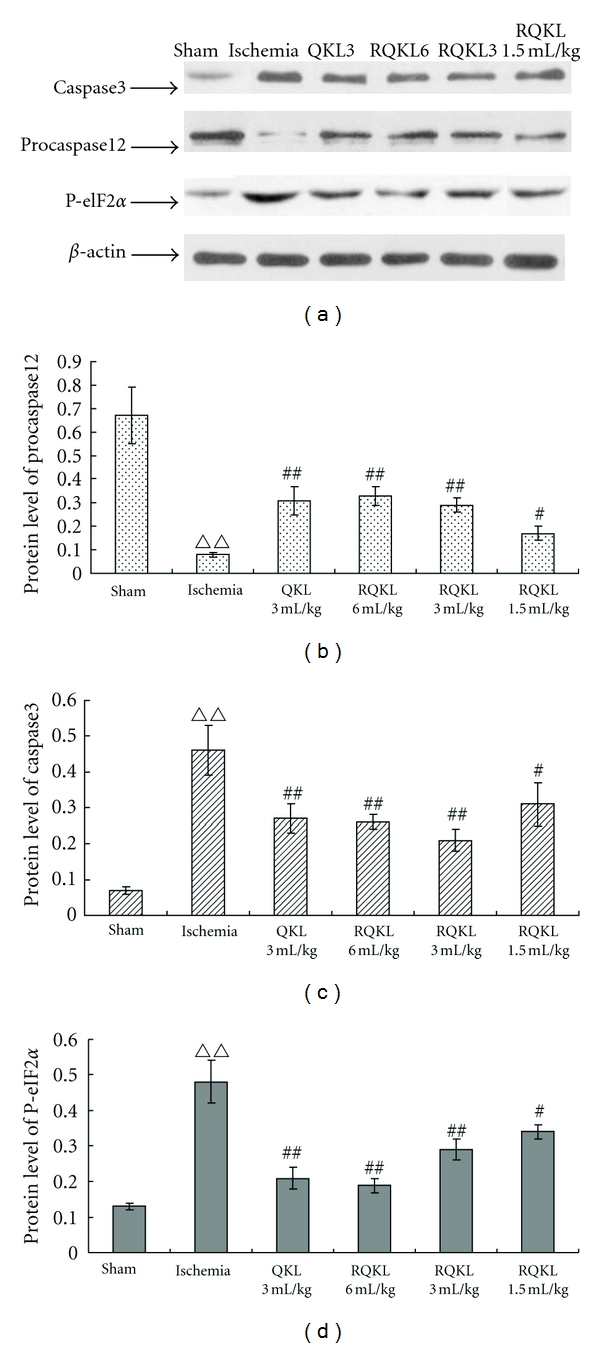
Effect of RQKL on protein levels of caspase3, procaspase12, and P-elF2a in cortex of MCAO mouse. Mice were subjected to 1.5 h ischemia followed 22.5 h reperfusion. QKL or RQKL was injected immediately after ischemia. The tissue samples were from the cerebral cortex. The panels are representative Western blotting analyses of caspase3, procaspase12 and P-elF2a. The gray values were calculated, and protein levels were expressed by the ratio of aim protein/*β*-actin as mean ± SEM. Notes ^ΔΔ^ *P* < 0.01, versus normal, ^##^ *P* < 0.01, versus ischemia, ^#^ *P* < 0.05, versus ischemia, *n* = 5. Primary antibodies were diluted, caspase12 1 : 400, caspase3 1 : 2000, and P-elF2a 1 : 500.

Caspase-3 is an important key protein in cell apoptosis. Moreover, it is well known that caspase-3 is induced after ischemic insults [39]. In this study, caspase-3 in the cortex increased markedly, whereas QKL and RQKL injections noticeably reduced the protein level (Figures 5(a) and 5(b)). Caspase-12 plays a role in apoptotic cell death by ER stress [20]. Therefore, we examined the effects of RQKL treatment on the induction of caspase-12 after ischemia. Western blotting analysis showed that caspase-12 was activated 24 h after hypoxia-ischemia, as evidenced by a decrease in the level of the procaspase-12, which was largely restored by QKL and RQKL (about 50% restoration compared with the model group) (Figures 5(a) and 5(c)). A key feature of ER stress induced by cerebral ischemia is the blocking of translation at the initiation step, as indicated by increased phosphorylation of eIF2*α* [40, 41]. Therefore, we examined whether RQKL affects the phosphorylation of eIF2*α*. The level of phospho-eIF2*α* in injured cortex was markedly increased 24 h after ischemia, whereas the injection of QKL or RQKL noticeably reduced the levels of phospho-eIF2*α* (approximately 30 to 60% reduction, compared with the model group) (Figures 5(a) and 5(d)).

#### 3.2.5. Antioxidative Effects of RQKL Injection

**Figure 6:**
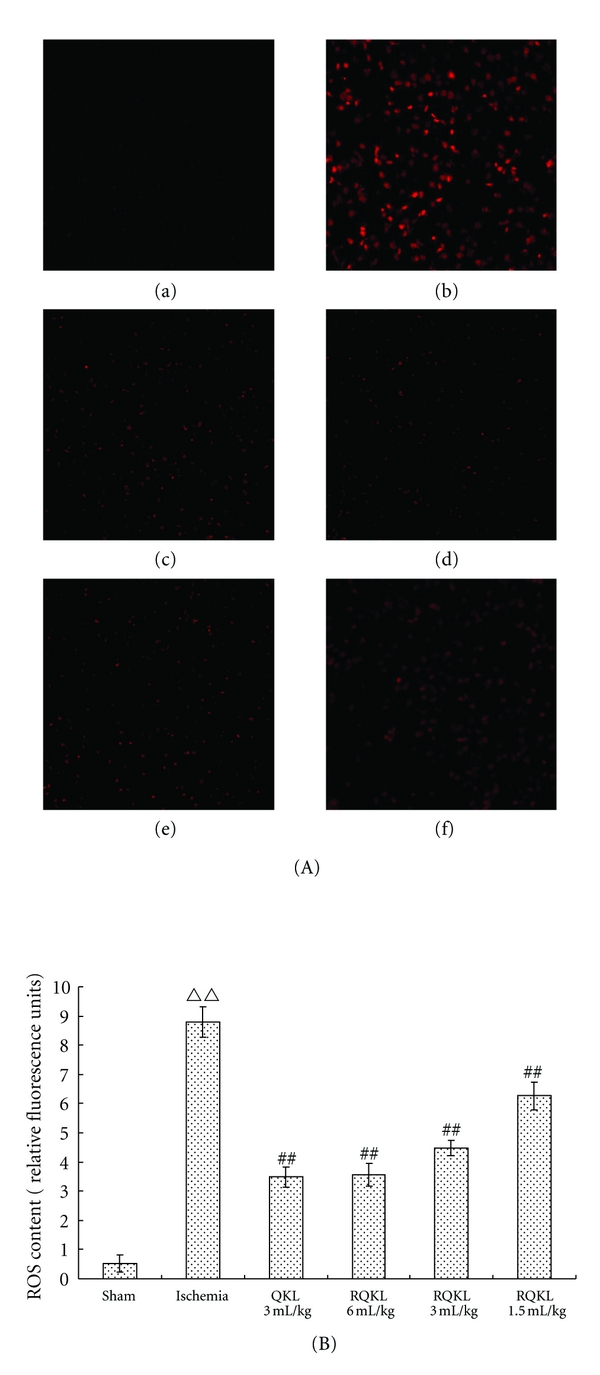
Effects of RQKL injection on reactive oxidative species in mice undergoing middle cerebral artery occlusion. Relative intensity of red fluorescence represents reactive oxygen species (ROS) content in the prefrontal cortex ((A) ×200) regions of the injured hemisphere. (a) sham, (b) ischemia, (c) ischemia+QKL3ml/kg, (d) ischemia+RQKL 6 mL/kg, e-ischemia+RQKL3ml/kg, (f) ischemia+RQKL1.5 mL/kg. Relative fluorescence intensity in five sites from one section was determined by fluorescence microscopy. The mean value of ROS content was calculated and expressed as mean ± SEM (B). ^ΔΔ^ *P* < 0.01, versus sham, ^##^ *P* < 0.01, versus ischemia, *n* = 5.

Reactive oxygen species production was detected using DHE staining. As shown in (Figures 6(A) and 6(B), no fluorescence was evident in sham mice brain. A large number of fluorescent nerve cells contributed to significantly enhanced fluorescence in the prefrontal cortex at 24 h after MCAO. Following treatment with QKL and RQKL, the number of fluorescent nerve cells was reduced, and the fluorescence intensity was weakened, indicating that RQKL reduced ROS production. Quantitative analysis showed that fluorescence in the cortex was significantly decreased in all the RQKL doses groups compared with the model group (*P* < 0.01, Figure 6(c)). This was consistent with our previous work that QKL injection has antioxidative effects [29].

#### 3.2.6. Intracellular Calcium

**Figure 7:**
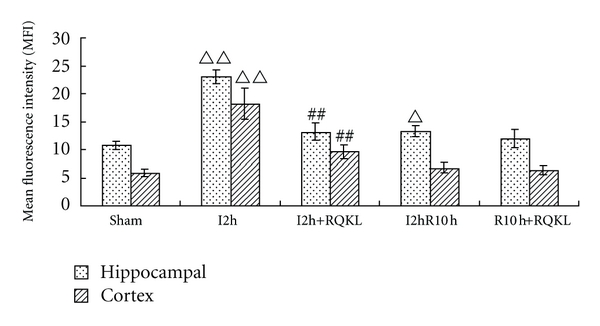
Effect of RQKL on intracellular Ca^2+^ in brain cells. Mice were divided into five groups: sham group, I2h group for cerebral ischemia 2 h, I2hR10h group suffering ischemia 2 h followed 10 h reperfusion. RQKL therapeutic group received RQKL (3 mL/kg) immediately after ischemia. Neurons of hippocampal and forehead cortex regions were detected by flow cytometry analysis. The results are expressed as mean fluorescence intensity (MFI). Note. ^ΔΔ^ *P* < 0.01, versus sham, ^Δ^ *P* < 0.05, versus sham, ^##^ *P* < 0.01, versus ischemia, *n* = 6.

As shown in Figure 7, intracellular Ca^2+^ markedly increased 2 h after ischemia, significantly compared with the sham group (*P* < 0.01), whereas the injection of RQKL noticeably reduced the Ca^2+^ content. However, for mice suffering 2 h ischemia following 10 h reperfusion, only intracellular Ca^2+^ of hippocampal cells increased, and RQKL did not significantly reduce it. So RQKL only performed modulation effect of intracellular calcium in the early period after ischemia.

## 4. Discussion

QKL injection is a famous Chinese medicine widely used in China for more than thirty years. However, since the National Accident Data Recorder Monitoring Center noticed the first case of anaphylaxis following administration of QKL in November 2001, medical journals have published some case reports about the adverse drug reactions and adverse events due to its use, so the safety of QKL injections became a focus of public opinion [9]. Much more attention has been attached to drug safety of traditional Chinese medicine (TCM), as well as efficiency. Redeveloping famous Chinese medicine formulas is a new way to improve traditional Chinese medicine, which is important for the modernization and globalization of TCM. Aimed at acute ischemic stroke, one of the indications of QKL, we refined QKL into a novel medicine, namely RQKL. It is composed of only four components of QKL, which has an advantage in quality stabilization and drug safety. In this study, we researched the efficiency and therapeutic time window of RQKL using MCAO mice, compared with QKL. The results showed that the injection of RQKL at doses of 1.5, 3, 6 mL/kg protected the brain from ischemic injury, as evidenced by reductions in infarct volume and neurological function. Most importantly, administration of RQKL provided a wide therapeutic window of 6 h.

The time window in which a drug is effective varies between drugs, commonly ranging between 2–4 h for ischemic stroke drugs [42–44], but occasionally extended to 12 h [45]. Administration beyond the therapeutic window can reduce or even abolish the pharmacodynamic action. This present study has defined the therapeutic window for RQKL injection in the treatment of brain ischemia. Incompatible with QKL, RQKL can also significantly reduce infarct volume and improved focal and general neurological function when administered in a broad time window after ischemia. RQKL injection at 6 h after ischemia significantly reduced infarct volume and improved neurological function, so the therapeutic window for RQKL injection for MCAO mice can be said to extend to over 6 h. This may be profited from multimechanisms of the compound Chinese medicine, for it showed remarkable effects of promoting endothelial nitric oxide synthase expression, reducing calcium overload, regulating matrix metallopeptidase 9 expression and inhibiting inflammation in a murine model of cerebral ischemia/reperfusion [5–8].

In this study, we found that RQKL can effectively suppress apoptosis *in vivo*. RQKL treatment significantly inhibited apoptosis under *in vivo* ischemic conditions, as indicated by the results of TUNEL assays. Moreover, the present findings indicated that the protective effect of RQKL on ischemic injury may be medicated in part by restoration of ER dysfunction. Previous studies showed that mitochondria play a central role in neurons apoptosis after ischemia. However, recent studies suggested that ER damage is involved in neuronal cell death induced by cerebral ischemia [23, 24, 46]. In the present study, we investigated the effect of RQKL on ER dysfunction under pathological conditions. Consistent with the results reported in recent years [18, 47], in mice subjected to 1.5 h ischemia and 22.5 h reperfusion, we observed an remarkable increase in the level of the eIF2*α*-phosphorylation in the ischemic cortex, which indicated that ischemia/reperfusion caused severe ER damage. On the other hand, treatment with RQKL significantly inhibited p-eIF2*α* induction. Therefore, the protective effects of RQKL in ischemia/reperfusion injury may be partly due to inhibition of ER stress and subsequent apoptotic signaling pathway.

ER stress-induced cell death has been shown to involve the activation of caspase-12, which subsequently activates executer caspases such as caspase-3 [20, 21, 48, 49]. Caspase-12 is specific to insults that elicit ER stress and is not proteolytically activated by other death stimuli [20]. Previous studies have shown that caspase-12 is activated after permanent and transient middle cerebral artery occlusion, and many caspase-12 positive cells exhibited DNA fragmentation; on the other hand, mice that are deficient in caspase-12 are more resistant to ER stress-induced apoptosis [20]. This indicated the activation of caspase-12 is involved in ischemia-induced apoptosis. We found caspase-12 was activated 24 h after MCAO and RQKL remarkably suppressed the activation, which indicated that RQKL inhibits caspase-12 dependent apoptotic pathway. Furthermore, RQKL also decreased caspase-3 level significantly. Caspase-3 activation is involved in caspase-12 mediated apoptotic cascade, while activated caspase-3 is directly responsible for DNA fragmentation [20, 21, 48]. It is possible that caspase-3 activation is inhibited by suppression of caspase-12. Thus, RQKL might inhibit caspase-3 activation by suppressing ER stress-mediated apoptotic signaling and therefore reduce the extent of apoptosis.

Studies suggested that multiple causes of ER stress occur in neurons following cerebral I/R: intracellular calcium homeostasis, aggregation of proteins, decreased protein degradation, and accumulation of ROS in ER and Golgi structures [50, 51]. Recent studies suggested that disruption of intracellular calcium homeostasis could induce ER stress and kill cells [52]. In this study, we found intracellular calcium raised markedly 2 h after ischemia; on the other hand, RQKL can significantly depress intracellular Ca^2+^. Like intracellular calcium homeostasis, ROS also played an important effect in causing ER stress [51, 53]. RQKL showed excellent antioxidative effects, evidenced by notably reducing ROS. So the inhibition effect to ER stress of RQKL may be immediate or mediated by anti-oxidative as well as intracellular calcium modulation. Considering the findings of the present study, we speculate that RQKL is a promising novel drug for acute ischemia stroke, and it has multiple neuroprotective effects, although the mechanism of its actions needs to be elucidated further.

## Abbreviations

MCAO: Middle cerebral artery occlusion
ROS: Reactive oxygen species
ER: Endoplasmic reticulum
RQKL: Refined Qingkailing.
